# К юбилею академика РАН Галины Афанасьевны Мельниченко

**DOI:** 10.14341/probl12855

**Published:** 2021-12-30

**Keywords:** Галина Афанасьевна Мельниченко, эндокринолог, юбилей

## Abstract

Галина Афанасьевна Мельниченко — известный российский ученый-эндокринолог, один из ведущих в стране, автор и руководитель фундаментальных и прикладных исследований. Г.А. Мельниченко — один из лидеров создания методологии по организации эндокринологической службы Российской Федерации. Г.А. Мельниченко является одним из ведущих специалистов Российской Федерации по изучению заболеваний щитовидной железы, гипоталамо-гипофизарной системы, орфанных эндокринопатий, надпочечниковой патологии, синдромов множественных эндокринных неоплазий.

## Abstract (en)

Galina A. Melnichenko is a famous Russian scientist-endocrinologist, one of the leading in the country, author and head of fundamental and applied research. G.A. Melnichenko is one of the leaders in creating a methodology for organizing the endocrinological service of the Russian Federation. Galina Melnichenko is one of the leading specialists in the Russian Federation in the study of diseases of the thyroid gland, hypothalamic-pituitary system, orphan endocrinopathies, adrenal pathology, multiple endocrine neoplasia syndromes.

Эндокринологическое общество 28 ноября 2021 г. обмечает юбилей замечательного ученого, академика РАН, заместителя директора по научной работе ФГБУ НМИЦ эндокринологии Минздрава России, вице-президента Российской ассоциации эндокринологов, блестящего клинициста и яркого педагога — Галины Афанасьевны Мельниченко.

Галина Афанасьевна Мельниченко — известный российский ученый-эндокринолог, один из ведущих в стране, автор и руководитель фундаментальных и прикладных исследований. Г.А.Мельниченко является одним из ведущих специалистов Российской Федерации по изучению заболеваний щитовидной железы, гипоталамо-гипофизарной системы, орфанным эндокринопатиям, надпочечниковой патологии, синдромам множественных эндокринных неоплазий.

В 1972 г. Г.А. Мельниченко окончила лечебный факультет РГМУ (МОЛГМИ им. Н.И. Пирогова). По окончании ординатуры и аспирантуры работала младшим научным сотрудником Института экспериментальной эндокринологии и химии гормонов АМН СССР (ныне ФГБУ «Эндокринологический научный центр» Минздрава России). С 1979 по 1987 г. была ассистентом, а затем доцентом на кафедре внутренних болезней №1 ММА им. И.М. Сеченова (ныне Первый МГМУ им. И.М. Сеченова, где в 1988 г. при 1-м лечебном факультете под руководством профессора Ивана Ивановича Дедова была создана кафедра эндокринологии). С 1992 г. Галина Афанасьевна — профессор кафедры эндокринологии ММА им. И.М. Сеченова, член-корреспондент РАМН с 2004 года, академик РАМН с 2011 года, академик РАН c 2013 года — Отделение медицинских наук. В 2002 г. Галина Афанасьевна назначена на должность директора Института клинической эндокринологии ФГБУ «Эндокринологический научный центр», с 2020г. и по настоящее время — заместитель директора Центра по науке.

Галина Афанасьевна — автор и руководитель многих фундаментальных и прикладных исследований в эндокринологии. Основные направления научной деятельности посвящены изучению проблем нейроэндокринной системы, вопросам персонифицированной диагностики и лечения заболеваний гипоталамо-гипофизарной системы, роли полиморфизма пролактина, клиническому значению его изоформ. Г.А. Мельниченко принадлежит разработка подходов к ведению пациентов с синдромом гиперпролактинемии, различными формами эндокринного бесплодия у мужчин и женщин.

Г.А. Мельниченко и ее учениками внедрены технологии диагностики и динамической стратификации папиллярного рака щитовидной железы, показания к использованию радиоактивного йода при раке щитовидной железы, развернуты широкомасштабные исследования по мониторингу йододефицитных состояний в Российской Федерации, результаты которых легли в основу Постановления правительства Российской Федерации и Национальной программы «О мерах профилактики заболеваний, связанных с дефицитом йода». Г.А. Мельниченко до настоящего времени является руководителем и научным куратором Национальной программы по скринингу, мониторингу и профилактике йододефицитных состояний в России.

Г.А. Мельниченко и ее ученикам по праву принадлежат приоритет и международное признание в области изучения функциональной системы, регулирующей половые функции у мужчин и женщин, в изучении возможности и разработке методов коррекции репродуктивных нарушений при различных эндокринопатиях, в том числе с использованием методом вспомогательных репродуктивных технологий, а также способы гормональной реабилитации мужчин и женщин в пожилом возрасте.

Академик РАН Г.А. Мельниченко — член Европейских ассоциаций по эндокринологии, эксперт ВАК Российской Федерации по терапевтическим дисциплинам, главный редактор журнала «Клиническая и экспериментальная тиреоидология», заместитель главного редактора журналов «Проблемы эндокринологии» и «Ожирение и метаболизм».

Галина Афанасьевна — автор более 800 научных публикаций, монографий, руководств, методических пособий, в том числе выдержавшего уже три издания и переведенного на ряд языков стран СНГ учебника по эндокринологии для студентов медицинских вузов, что было отмечено премией Правительства Российской Федерации 2021 в области образования за учебник «Эндокринология».

Г.А. Мельниченко является куратором разработки более 30 клинических рекомендаций, выпущенных Российской ассоциацией эндокринологии, в том числе в соавторстве с ассоциациями онкологов, радиологов, офтальмологов и др., а также является руководителем исследований, выполняемых по грантам Российского научного фонда, РФФИ.

Галина Афанасьевна Мельниченко является членом Бюро Отделения медицинских наук РАН; членом Президиума Высшей аттестационной комиссии РФ по терапевтическим дисциплинам; почетным членом академии наук Казахстана; вице-президентом Российской ассоциации эндокринологов; членом Европейской ассоциации нейроэндокринологов, Европейской тиреоидологической ассоциации, Международной ассоциации эндокринологов (Endo-society). В 2008 г. награждена орденом почета Российской Федерации за вклад в науку. В 2011 г. академику РАН Мельниченко Г.А. присвоена первая премия Всероссийского конкурса «Призвание» в номинации «За создание нового направления в медицине».

Галина Афанасьевна — талантливый организатор, невероятно творческий человек, достойный пример для коллег и учеников. Галину Афанасьевну всегда отличают многогранность мышления, результативность в клинической деятельности, беззаветная преданность выбранной профессии и специальности, высочайший профессионализм, самоотдача, стремление к совершенству, принципиальность и дипломатичность одновременно, утонченный ум и потрясающее чувство юмора — все это мы с гордостью говорим и пишем о Вас, дорогая и глубокоуважаемая Галина Афанасьевна!

Коллеги, ученики, читатели и редакция журналасердечно поздравляют Галину Афанасьевну с юбилеем,желают здоровья и новых творческих свершений.

